# Feasibility and practicality of a novel teaching aid for microvascular anastomosis simulation training in neurosurgery generated by 3D printing

**DOI:** 10.3389/fsurg.2025.1546573

**Published:** 2025-04-30

**Authors:** Guosheng Shi, Huiling Ren, Dawei Zhao, Yunwei Cui, Xiang Su, Suwei Yan, Wei Bu

**Affiliations:** ^1^ Department of Neurosurgery, The Third Hospital of Hebei Medical University, Shijiazhuang, China; ^2^ Department of Neurology, The Third Hospital of Hebei Medical University, Shijiazhuang, China; ^3^ Institute of Bone Research, The Third Hospital of Hebei Medical University, Shijiazhuang, China

**Keywords:** 3D printing, microsurgery, vascular anastomosis, simulation training, 3D printed teaching aid, neurosurgery

## Abstract

**Background:**

This study aimed to develop a novel teaching aid for microvascular anastomosis training in neurosurgery using 3D printing technology based on CT and MRI imaging data, and to evaluate its effectiveness and practicality.

**Methods:**

Based on CT or MRI imaging data, a 3D model integrating micro-vessels, skull, and brain tissue was fabricated and connected to a peristaltic pump and a pipeline system to create a teaching aid for microvascular anastomosis simulation training. Twenty senior medical students were recruited and divided into two groups: a control group, which trained using traditional soft rubber tubes, and an observation group, which trained using the 3D-printed teaching aid. Following the training, participants from both groups performed chicken wing artery anastomosis. The training outcomes, including the patency rate of vascular anastomosis, the time required to complete the anastomosis, and the trainees' surgical performance, were evaluated. Additionally, six experienced neurosurgeons were recruited to teach the course using both teaching aids for two hours each. They were then surveyed via a questionnaire to assess and rate the effectiveness of the teaching aids.

**Results:**

The observation group demonstrated a significantly higher patency rate of vascular anastomosis, a shorter time to complete the anastomosis, and higher scores for surgical proficiency and procedural standardization compared to the control group (all *P* < 0.001). Additionally, the neurosurgeons provided positive evaluations of the novel 3D-printed teaching aid, awarding high scores for its practicality, scientific rigor, and overall effectiveness.

**Conclusion:**

The novel 3D-printed teaching aid serves as an effective tool for microvascular anastomosis training in neurosurgery, offering significant advantages such as enhanced training effectiveness, high-fidelity simulation, cost efficiency, and customization capabilities.

## 1 Introduction

Three-dimensional (3D) printing has revolutionized medical education by converting clinical imaging data into anatomically precise models through additive manufacturing. This technology utilizes biocompatible polymers to create layered constructs that replicate both normal anatomy and pathological conditions based on Digital Imaging and Communications in Medicine (DICOM) standards, effectively linking diagnostic imaging with surgical skill development (1).

In neurosurgical training, patient-specific 3D-printed models offer four distinct advantages compared to traditional cadaveric methods: (1) improved tactile feedback through multi-material fabrication mimicking tissue elasticity (Young's modulus: 2–35 kPa); (2) durability allowing over 50 reuse cycles; (3) avoidance of ethical concerns regarding biological specimens; and (4) reduced operational costs (2). Clinical studies demonstrate that incorporating these models into surgical education enhances critical performance indicators, including reduced operative time and improved anatomical recognition accuracy (3).

Micro-neurosurgical procedures are among the most complex surgical operations, requiring exceptionally refined surgical skills (4). While live animal models are considered one of the most effective methods for practicing these skills, their widespread use is limited by ethical concerns, high costs, and limited availability of animal resources (5). Therefore, the aim of this study was to develop a novel, low-cost, reusable, and scientifically validated teaching aid for micro-neurosurgical training using 3D printing technology and imaging modalities such as CT and MRI. Specifically, the study focused on creating a teaching aid model that integrates the skull, brain tissue, and blood vessels for microvascular anastomosis training. This was achieved by converting clinical imaging data into 3D spatial models through layer-by-layer material deposition. The feasibility and effectiveness of this teaching aid for microvascular anastomosis training were then evaluated.

## 2 Methods

### 2.1 Preparation of a novel 3D printed teaching aid

#### 2.1.1 3D modeling

Typical cases were selected, their CT data in DICOM format with a slice thickness of 1 mm were acquired. Imaging data of blood vessels, skull, and brain tissue were obtained. Then digital 3D model was constructed using Rhino software (Robert McNeel & Assoc USA) (6).

#### 2.1.2 Slicing

After the 3D model was constructed, the model was processed with the slicing software (Cura 4.4.1, Ulitmater, USA), i.e., (7), the 3D model was ***sliced*** into several slices with required thickness according to a certain coordinate axis, which was then transformed into cross-sectional information that can be read by 3D printer (8).

### 2.2 3D printing and spraying

**Figure 1:**
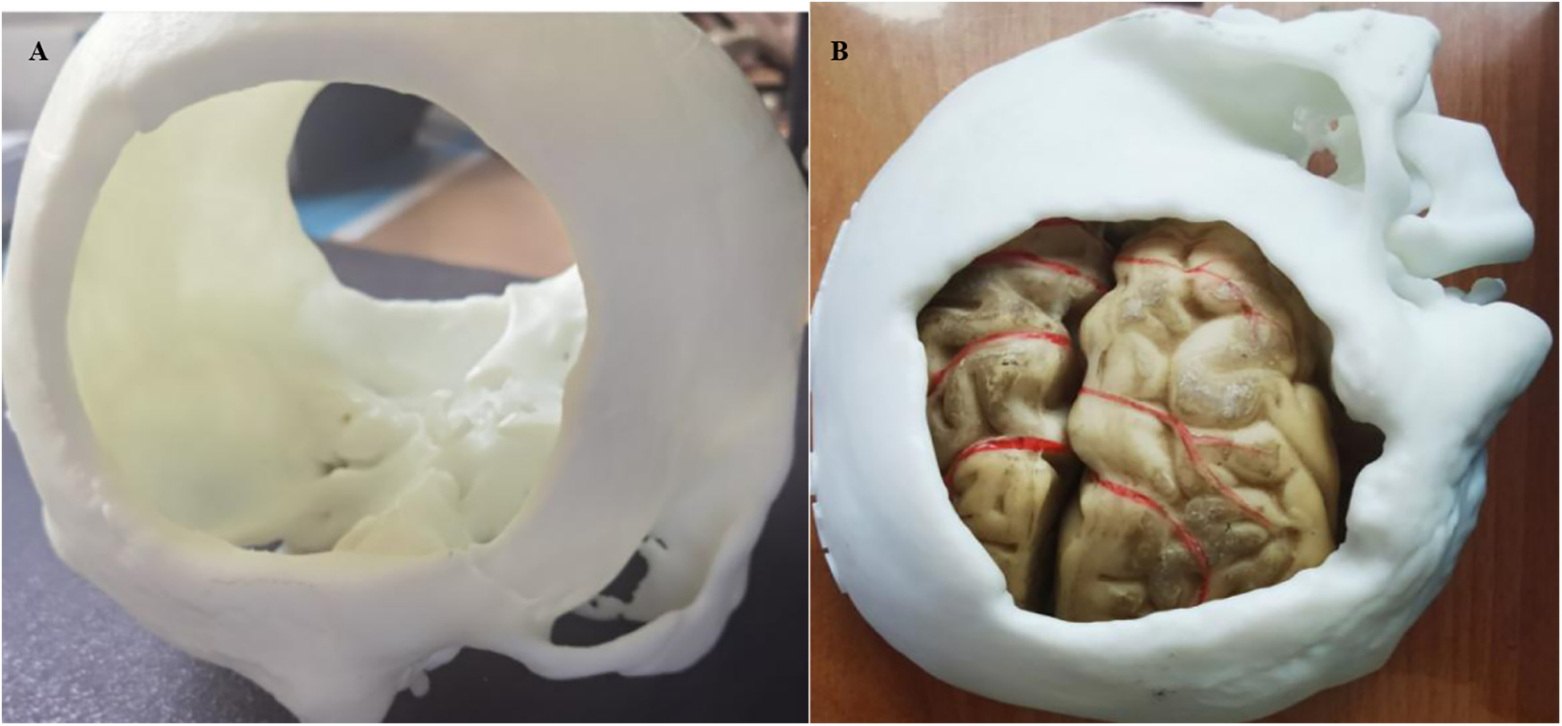
**(A)** 3d printed skull structure. **(B)** Complete anatomical model combining multiple tissues (skull, brain tissue, and blood vessels) together.

The 3D-printed artificial blood vessels employ hydrogel-based biocomposite materials, demonstrating structural parameters with wall thickness ≥0.4 mm, internal diameter ≥0.8 mm, and customizable length dimensions. These engineered constructs exhibit mechanical properties within the following ranges: tensile strength 0.5–5 MPa, Shore 00 hardness 5–30 degrees, and puncture resistance 5–20 kN/m. The biomechanical characteristics of these 3D-printed hydrogel vascular grafts closely approximate those of native blood vessels, particularly in terms of mechanical strength matching and realistic tactile simulation. This technological advancement provides an optimized training platform for microvascular anastomosis techniques, offering superior biomimetic performance in surgical education applications. The material system achieves an optimal balance between structural integrity and biological compliance, fulfilling both instructional requirements and biomechanical simulation criteria (9, 10). After the printer reads the cross-sectional information of the 3D model, the 3D printing materials and adhesive materials were stacked layer by layer in the 3D space by hot melt extrusion. The object was then formed after cooling (11) (Figure 1).

### 2.3 Design of teaching aids

**Figure 2:**
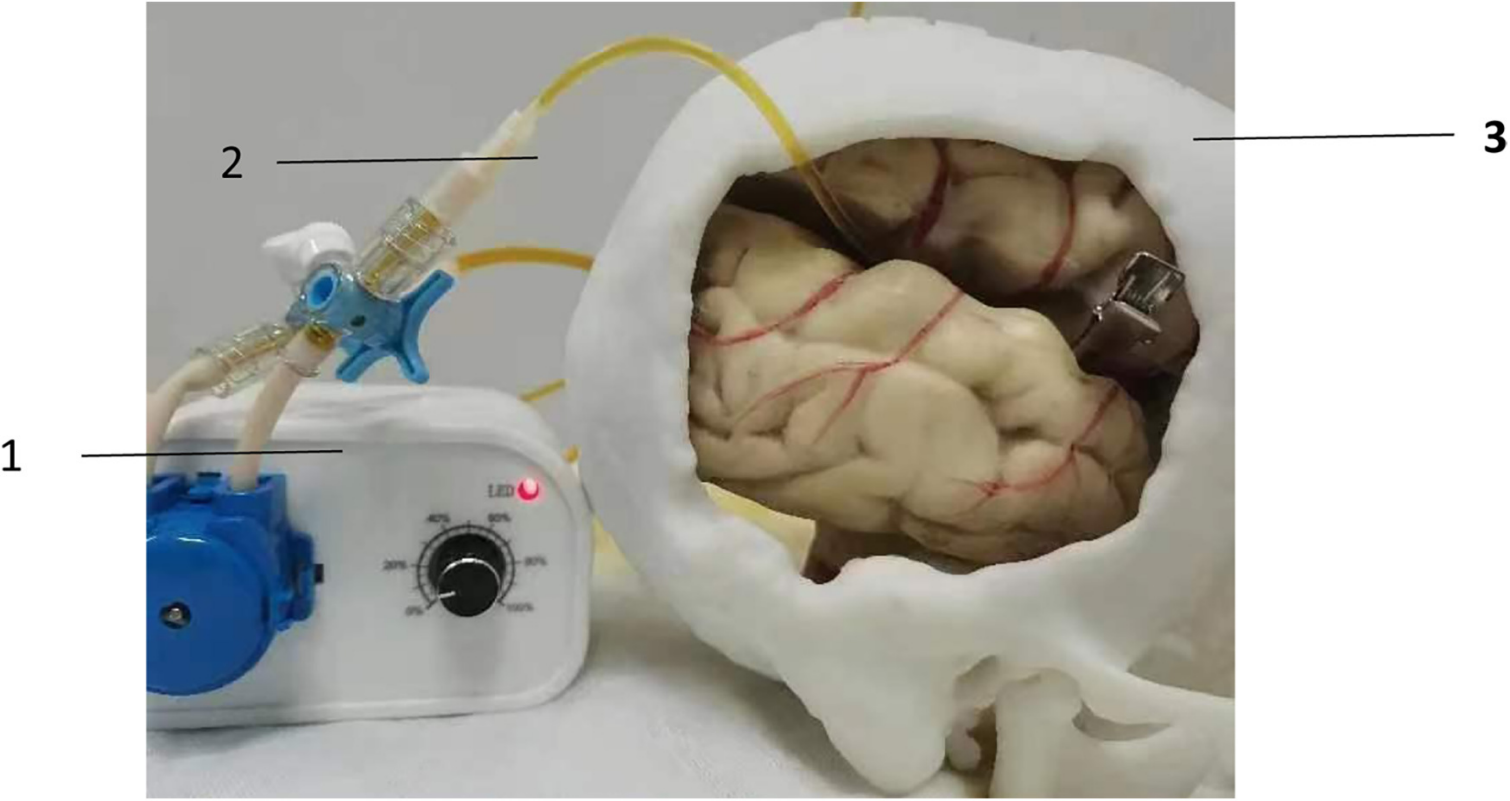
Evaluation of suture effect using peristaltic pump. (1) peristaltic pump; (2) pipeline system; (3) 3D printed solid model.

The teaching aid for microvascular anastomosis in neurosurgery is composed of a solid model, a peristaltic pump, and a pipeline system. With the use of 3D printer and CT or MRI imaging technology, the solid model was generated by combining bony structures, soft tissues, and blood vessels at the lesion sites of common neurosurgical diseases that were printed using different materials together in a 1:1 ratio according to the actual human anatomy. This model can simulate surgical procedures, and allow trainees to practice vascular suturing after removal of the corresponding bone flap or other tissues in the brain. After completion of suturing, suturing effect can be evaluated by connecting the micro-vessels inside the teaching aid to the pipeline system, and pumping colored liquid into the pipeline through the peristaltic pump (Figure 2).

### 2.4 Evaluation of the effectiveness of the teaching aid

#### 2.4.1 Training effect

A total of 20 senior undergraduate medical students (with a surgical experience of <10 cases) from Hebei Medical University were recruited, randomly and double blindly divided into a control group and an observation group, with 10 trainees in each group. Blinded selection protocol: Step 1: we number participants as 1–20. Step 2: Determine the ratio of participants in the experimental group and control group For 20 participants, 10 will go to each group. Step 3: Excel software was used for random grouping of 20 digits. All grading instructors did not participate in the arranging and teaching of any student group.

**Figure 3:**
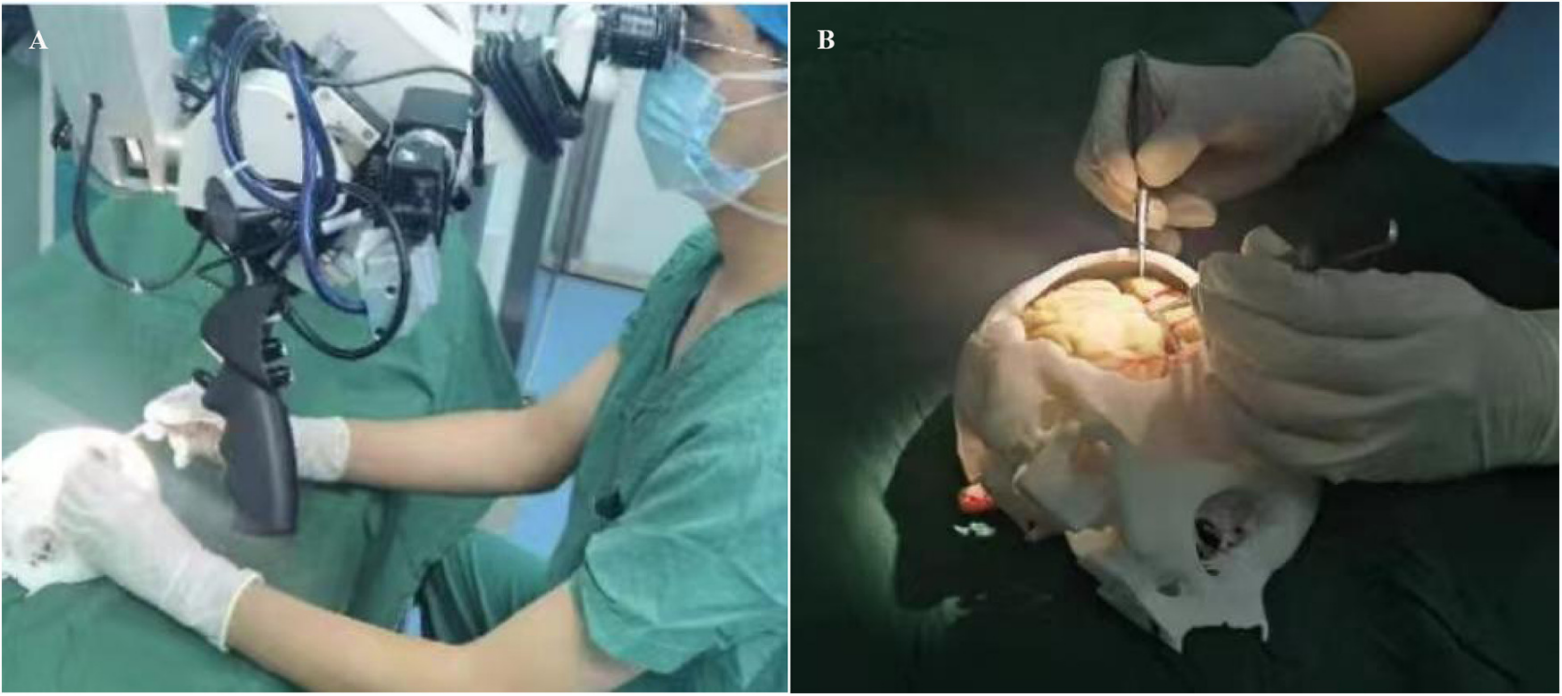
Actual use of the novel 3D printed teaching aid by trainees to practice vascular suturing. **(A)** demonstrates a trainee performing microvascular anastomosis technique during an instructional exercise. **(B)** illustrates the training simulator under surgical microscope visualization.

In the control group, traditional soft rubber tube was used as teaching aids for microvascular anastomosis training. In the observation group trainees were trained using the novel 3D-printed teaching aid. Training was performed for a total of 14 days (1 h per day). After training, trainees performed anastomosis of the chicken wing artery (Figure 3). The anastomosis of the middle artery in the deep part of the chicken wing tests the trainees' operational skills under limited surgical vision, which is widely adopted in microsurgical training programs for mastering vascular anastomosis procedures. The simulated blood vessels used for teaching and validation showed no significant differences in vessel diameter among the groups. After completion of chicken wing artery anastomosis, the following indicators were examined: (1) the patency rate of vascular anastomosis; (2) time taken to complete the anastomosis (min); (3) trainees' performance, i.e., surgical proficiency and standardization, was evaluated and scored in a blinded manner by 6 teachers with rich clinical teaching experience. Scores range from 1.0–10.0 where higher scores indicate high surgical proficiency. The average scores were taken.

After completion of the examination, the 20 trainees were surveyed with a questionnaire to rate the novel 3D-printed and traditional teaching aids. The questionnaire include the following items: (1) learning motivation: whether the use of the teaching aids could promote trainees' motivation to practice the procedures; (2) clinical similarity: whether the shape and composition of the teaching aids are similar to actual clinical practice; (3) trainees' satisfaction: whether the teaching aids are reasonably designed, easy to use, and can be widely used in clinical training. Scores range from 1.0–5.0 where higher scores indicate high ratings of the teaching aid given by trainees.

#### 2.4.2 Teaching efficacy

Six neurosurgeons with rich clinical experience from the Affiliated Hospital of Hebei Medical Universit were recruited. They taught the course with the used of novel 3D printed teaching aid and traditional teaching aid for 2 h, respectively. Then evaluation was conducted using a questionnaire including the following items: (1) practicality: whether the teaching aids could be used in neurosurgical teaching; (2) scientificity: whether the design of the teaching aid is reasonable and whether it can realistically reproduce clinical situations; (3) effectiveness: whether the teaching aids can enable trainees to master micro-neurosurgical skills. Scores range from 1.0–5.0 where higher scores indicate high ratings of the teaching aid given by neurosurgeons.

### 2.5 Statistical analysis

All analyses were performed using the SPSS software 20.0. Continuous variables were expressed as mean ± standard deviation (SD), and *t*-test was used for comparison between groups. Categorical variables were expressed as percentages (%), and *χ*2 test was used for comparison between groups. *P* < 0.05 was considered as statistically significant.

## 3 Results

### 3.1 Comparison of training effect between two groups

The observation group showed significantly higher patency rate of vascular anastomosis (0.87 ± 0.05 vs. 0.69 ± 0.06), shorter time taken to complete the anastomosis (15.32 ± 1.23 vs. 19.32 ± 1.34), and higher scores for trainees' performance (8.62 ± 0.64 vs. 7.89 ± 0.15) compared with the control group (all *P* < 0.001), indicating that the effect of the novel 3D printed teaching aid for microvascular anastomosis training was significantly better than that of traditional soft rubber tube.

Ratings of the teaching aids for microvascular anastomosis given by the trainees showed that the scores for learning motivation, clinical similarity, and satisfaction were 4.6, 4.3, 4.5, respectively, in the observation group, which was 3.1, 2.6, 3.2, respectively, in the control group, suggesting that the use of the novel 3D-printed teaching aid was highly rated by trainees when compared to the use of traditional teaching aid.

### 3.2 Teaching effect

Ratings of the teaching aids given by 6 neurosurgeons showed that teaching with the use of novel 3D-printed teaching aid scored significantly higher in practicality, scientificity, effectiveness than teaching with the use of traditional teaching aid (practicality score: 4.31 ± 0.34 vs. 3.18 ± 0.53); scientificity score: 4.56 ± 0.56 vs. 3.45 ± 0.33; effectiveness score: 4.45 ± 0.25 vs. 3.03 ± 0.56, all *p* < 0.001). The results suggest that neurosurgeons rated the novel 3D printed teaching aid positively, with better practicality, scientificity, and effectiveness.

## 4 Discussion

### 4.1 The effect of application of the novel 3D printed teaching aid

3D-printed surgical training models significantly enhance vascular anastomosis practice by replicating anatomical structures with high fidelity, simulating intraoperative visualization, and recreating clinically realistic scenarios. Unlike traditional rubber-based training tools, 3D-printed surgical models offer three key innovations that enhance anatomical fidelity and clinical relevance.

The model is more in line with human anatomy; Realistic Surgical Angles: Exposure angles (45–60°) replicating standard pterional craniotomy approaches; Vessel Simulation: Dynamic physiological behaviors, including compliant vessel walls (0.12 ± 0.03 mm²/mmHg) and pulsatile blood flow (peak velocity: 15–25 cm/s). These advancements enable trainees to practice under lifelike surgical conditions, improving technical skill development and engagement. Additionally, instructors can demonstrate procedural techniques more effectively compared to conventional static resources like PowerPoint slides or textbook illustrations, strengthening educational outcomes (12).

Traditional rubber-based models lack clinical relevance due to oversimplified designs that poorly mimic real vascular anastomosis (13). While live animal models remain the gold standard for surgical training, their use is limited by ethical constraints, high costs, and operational complexity. In contrast, 3D printing converts clinical imaging data into anatomically precise, reusable models through layer-by-layer fabrication, offering cost-effective and ethically unconstrained alternatives (14). The 3D-printed neurosurgical simulator integrates region-specific anatomy, diverse vessel morphologies (15), and a pulsatile flow system (*via* peristaltic pump) for anastomosis patency testing. Its modular design—combining instructional guidance, hands-on training, and performance evaluation—creates a versatile platform that improves trainees' surgical proficiency, anatomical understanding, and procedural confidence (12).

In a review on microsurgical training (2), Chan WY et al. reported that the validation of microsurgery models is critical. The development of new technologies drives the invention of models for microsurgical procedures; however, most lack proper validation. This study designed a control group and an experimental group to undergo standardized microsurgical training courses using different models. The effectiveness was evaluated through expert subjective assessments and standardized objective validation, demonstrating validity in terms of content validity, construct validity, concurrent validity, and predictive validity.

### 4.2 Advantages and shortcomings of the noval 3D printed teaching aid

The results of this study demonstrate that the 3D-printed surgical training model significantly improved trainees' operative performance, receiving high satisfaction ratings from both trainees and experienced neurosurgeons. This enhanced efficacy can be attributed to four key advantages:

Clinical simulation: The model's anatomically complete structure, reconstructed from CT/MRI data via 3D printing, enables realistic replication of surgical field visualization and complex vascular anastomosis procedures within an operative window, achieving clinical fidelity (16). (2) Multi-caliber vessel designs for tailored suture training; Integrated pulsatile flow validation (*via* peristaltic pump and pipeline system) to assess anastomosis quality (17). (3) Increased learner engagement. As an innovative pedagogical tool, the model stimulates trainee motivation and satisfaction rates in training evaluations. (4) The model offers: Reusable components with mass-producible vascular units (18); 60% lower cost compared to traditional rubber vessel models (particularly for small-caliber.

While this study demonstrates the feasibility of 3D-printed vascular phantoms for educational applications, some notable limitations should be acknowledged. Firstly, the current models do not incorporate biomechanical stress simulations, which limits their ability to replicate the dynamic responses of vascular tissues under physiological pressures. Secondly, although the materials selected approximate the visual and tactile properties of real vasculature, discrepancies in haptic feedback compared to actual surgical scenarios remain. These limitations primarily stem from technical constraints in multi-material 3D printing and the complexity of simulating dynamic hemodynamic forces within static models. Future iterations could explore hybrid approaches combining 3D-printed scaffolds with embedded sensor arrays and hydrogel matrices to better simulate biomechanical stresses. Advancements in smart materials may also help bridge the haptic discrepancy gap identified in this study. Additionally,current clinical-grade 3D printers lack the resolution to accurately reproduce submillimeter microvasculature and neural tissue.Model accuracy is contingent on high-resolution CT/MRI data (0.5 mm slice thickness recommended); (19, 20).

### 4.3 Application prospects of 3D printing technology in neurosurgery education

In recent years, 3D printing technology has advanced rapidly. With continuous technological innovations, the cost of 3D printing has gradually decreased, while its ability to simulate realistic models has significantly improved (21). As 3D printing technology becomes increasingly mature, it is being utilized across a wide range of fields (16), including the production of medical models (22), implantable devices (23), artificial joints, and dental prosthetics (24). Micro-neurosurgical operations are among the most complex surgical procedures, requiring highly refined skills and extensive practice to master delicate techniques. Innovations in medical education have reduced the time needed for surgical training, and 3D-printed models offer significant potential as clinical teaching tools due to their advantages in personalization and on-demand customization. Visualization and simulation through 3D printing make clinical teaching and surgical practice more intuitive and realistic (25).

With ongoing advancements in 3D printing materials and technologies, the application of 3D printing in the field of micro-neurosurgical clinical education holds extensive promise.

## 5 Conclusions

The novel 3D printed teaching aid can be used as a good tool for microvascular anastomosis training in neurosurgery, due to its advantages of better training effectiveness, high realistic simulation, low cost, and personalization.

## Data Availability

The original contributions presented in the study are included in the article/Supplementary Material, further inquiries can be directed to the corresponding author.
